# Bayesian deterministic decision making: a normative account of the operant matching law and heavy-tailed reward history dependency of choices

**DOI:** 10.3389/fncom.2014.00018

**Published:** 2014-03-04

**Authors:** Hiroshi Saito, Kentaro Katahira, Kazuo Okanoya, Masato Okada

**Affiliations:** ^1^Department of Complexity Science and Engineering, Graduate School of Frontier Sciences, The University of TokyoKashiwa, Japan; ^2^Center for Evolutionary Cognitive Sciences, The University of TokyoTokyo, Japan; ^3^RIKEN Brain Science InstituteWako, Japan; ^4^Okanoya Emotional Information Project, Exploratory Research for Advanced Technology (ERATO), Japan Science and Technology AgencyWako, Japan; ^5^Department of Life Sciences, Graduate School of Arts and Sciences, The University of TokyoTokyo, Japan

**Keywords:** decision making, operant matching law, Bayesian inference, dynamic foraging task, heavy-tailed reward history dependency

## Abstract

The decision making behaviors of humans and animals adapt and then satisfy an “operant matching law” in certain type of tasks. This was first pointed out by Herrnstein in his foraging experiments on pigeons. The matching law has been one landmark for elucidating the underlying processes of decision making and its learning in the brain. An interesting question is whether decisions are made deterministically or probabilistically. Conventional learning models of the matching law are based on the latter idea; they assume that subjects learn choice probabilities of respective alternatives and decide stochastically with the probabilities. However, it is unknown whether the matching law can be accounted for by a deterministic strategy or not. To answer this question, we propose several deterministic Bayesian decision making models that have certain incorrect beliefs about an environment. We claim that a simple model produces behavior satisfying the matching law in static settings of a foraging task but not in dynamic settings. We found that the model that has a belief that the environment is volatile works well in the dynamic foraging task and exhibits undermatching, which is a slight deviation from the matching law observed in many experiments. This model also demonstrates the double-exponential reward history dependency of a choice and a heavier-tailed run-length distribution, as has recently been reported in experiments on monkeys.

## 1. Introduction

Does the brain play dice? This is a controversial question about the underlying processes of the brain in making a choice from several alternatives: Does the brain decide deterministically with some internal decision variables? Or does it calculate the probability of choosing individual alternatives and cast a “biased die” (Sugrue et al., 2005)? The former strategy is suggested according to our everyday experience. However, it is possible to think that choices emerge probabilistically by observing a sequence of decisions in a repetitive task. Herrnstein conducted a foraging experiment where a pigeon was placed into a box that was equipped with two keys and when a key was pressed it was rewarded with concurrent variable-interval schedules. He found a relationship between rewards and choices known as the “operant matching law” (Herrnstein, 1961). The law states that the fraction of the number of times one alternative is chosen against the total number of choices matches the fraction of the cumulative reward obtained from the alternative against the total reward. Behaviors satisfying the law have been observed in a variety of task paradigms and across species (de Villiers and Herrnstein, 1976; Gallistel, 1994; Anderson et al., 2002). Several learning models have been proposed to account for matching behavior (Corrado et al., 2005; Lau and Glimcher, 2005; Loewenstein and Seung, 2006; Soltani and Wang, 2006; Sakai and Fukai, 2008a; Simen and Cohen, 2009). These models have a commonality in that a model learns the probabilities of choosing each alternative directly, and then a choice is made stochastically. However, it is yet unknown whether matching behaviors can be accounted for by a deterministic model.

Here, we propose deterministic Bayesian decision making models for a two-alternative choice task. Our models stand on the incorrect but conceivable postulate that animals have a belief that the choice made in one trial does not affect a reward in subsequent trials. The models estimate the unknown reward probabilities for each alternative and deterministically choose the alternative that has the highest reward probability according to the *winner-take-all* principle. We first study a model with belief that the environment does not change. Note that this is an extension of the fixed belief model (FBM) (Yu and Cohen, 2009) for the two-alternative choice task. We demonstrate that this model satisfies the matching law in a steady state in static foraging tasks, in which reward baiting probabilities are fixed, but not in dynamic foraging tasks, in which the reward baiting probabilities change abruptly. Then, we devise two models that forget past experience and exhibit matching behaviors even in dynamic tasks. Moreover, these models can explain *undermatching*, which is a phenomenon observed across different species (Baum, 1974; de Villiers and Herrnstein, 1976; Baum, 1979; Gallistel, 1994; Anderson et al., 2002; Sugrue et al., 2004; Lau and Glimcher, 2005). We test these models by comparing their predicted reward history dependencies and run-length distributions to those seen in a monkey experiment.

## 2. Results

We studied deterministic Bayesian decision making models that demonstrated matching behaviors in a foraging task. The foraging task is a decision making task that simulates a foraging environment where an animal chooses one out of several foraging alternatives. There are two alternatives in this study although our results do not depend on this. We employed discrete trial-to-trial tasks that have often been used in recent experiments (Sugrue et al., 2004; Corrado et al., 2005; Lau and Glimcher, 2005). Each alternative has binary baiting state *f*_*i*_ (*i* ∈ {1, 2} is the index of an alternative), where *f*_*i*_ = 1 if a reward is baited and *f*_*i*_ = 0 otherwise. If *f*_*i*_ = 0, a reward is baited (*f*_*i*_ = 1) at the beginning of each trial by baiting probability λ^*t*^_*i*_, where *t* represents the number of the trial. If the baiting probabilities are fixed across trials, the task is called a *static* foraging task, otherwise it is called a *dynamic* foraging task (Sugrue et al., 2004). Suppose that *r*^*t*^_*i*_ indicates whether a subject receives a reward (*r*^*t*^_*i*_ = 1) or not (*r*^*t*^_*i*_ = 0), and *c*^*t*^_*i*_ indicates whether the subject chooses alternative *i* (*c*^*t*^_*i*_ = 1) or not (*c*^*t*^_*i*_ = 0) in trial *t*. When the subject chooses a baited alternative, i.e., *f*_*i*_ = 1 and *c*^*t*^_*i*_ = 1, the baited reward is consumed (*f*_*i*_ ← 0). This reward schedule is known as a “concurrent variable-interval schedule”(Baum and Rachlin, 1969).

Whichever alternative the subject chooses in the foraging task, the choice can affect the reward probabilities of alternatives in the future. Therefore, the optimal strategy is not to exclusively choose the foraging alternative that has the highest baiting probability. A behavioral strategy obeying the matching law is known to be nearly optimal for this task (Baum, 1981). Formally, the law states that

(1) $$\frac{{\bar{R}}_{i}^{t}}{\sum_{j}{\bar{R}}_{j}^{t}}=\frac{{\bar{C}}_{i}^{t}}{\sum_{j}{\bar{C}}_{j}^{t}},$$

where *R*^*t*^_*i*_ and *C*^*t*^_*i*_ correspond to the total reward obtained from alternative *i* and the number of choices of alternative *i* until trial *t*. It is known that human and animal behaviors in these kinds of tasks are well described by the generalized matching law (Baum, 1974)

(2) $$\log({\bar{R}}_{1}^{t}/{\bar{R}}_{2}^{t})=s\log({\bar{C}}_{1}^{t}/{\bar{C}}_{2}^{t})+\log k,$$

where *s* is sensitivity and *k* is bias. Equation (2) is equivalent to (1) if both *s* and *k* are unities.

### 2.1. Simple bernoulli estimators

First, we studied a simple normative Bayesian decision making model to clarify the underlying feasible computation for matching behaviors. Suppose that a subject makes a decision simply depending on its estimates of the reward probabilities for the alternatives. The estimate can be formally described as

(3) $$P_{i}^{t+1}=p(r_{i}^{t+1}=1|R^{t},C^{t}),$$

where *R*^*t*^ is a list of reward vectors *r*^*t*^ = (*r*^*t*^_1_, *r*^*t*^_2_) from trials 1 to *t* and *C*^*t*^ is a list of choice vectors *c*^*t*^ = (*c*^*t*^_1_, *c*^*t*^_2_) from trials 1 to *t*. The model employs a *winner-take-all* (WTA) strategy, i.e., it chooses the alternative that has the highest *P*^*t*^_*i*_. The model requires an assumption about a reward assignment mechanism to estimate *P*^*t*+1^_*i*_. One simple and conceivable assumption is that a choice is rewarded according to hidden reward probability μ^*t*^_*i*_ that is irrelevant to the past reward and choice history, i.e., *p*(*r*^*t*^_*i*_ = 1) = μ^*t*^_*i*_. This assumption is incorrect for our tasks but we have assumed that the model employs it and predicts μ^*t*^_*i*_ by Bayesian inference. Hence, *P*^*t*+1^_*i*_ is given by the predictive distribution over μ^*t*^_*i*_:

(4) $$P_{i}^{t+1}=\int_{0}^{1}d\mu \mu p(\mu_{i}^{t+1}=\mu |R^{t},C^{t}).$$

Note that *p*(μ^*t* + 1^_*i*_ = μ|*R*^*t*^, *C*^*t*^) can include a model's belief about the change of μ^*t*^_*i*_ in between trials. Our first model assumes that μ^*t*^_*i*_ is time invariant, i.e., *p*(μ^*t* + 1^_*i*_ = μ|*R*^*t*^, *C*^*t*^) = *p*(μ^*t*^_*i*_ = μ|*R*^*t*^, *C*^*t*^). The posterior distribution for an alternative is not updated if the alternative is not chosen. If it is chosen, the posterior distribution is updated

(5) $$\begin{array}{l} p(\mu_{i}^{t}=\mu |R^{t},C^{t})\propto p(r_{i}^{t}|\mu_{i}^{t}=\mu )p(\mu_{i}^{t-1}=\mu |R^{t-1},C^{t-1})\\ \;=\mu^{r_{i}^{t}}{\left(1-\mu \right)}^{1-r_{i}^{t}}p(\mu_{i}^{t-1}=\mu |R^{t-1},C^{t-1}). \end{array}$$

We employ the Beta prior, *p*(μ^0^_*i*_ = μ) = Beta(μ|a, b), which is a conjugate for the likelihood. Note that we set the hyper-parameters, *a* = *b* = 1, to make the prior non-informative in all simulations. Therefore, the posterior becomes a Beta distribution:

(6) $$p(\mu_{i}=\mu |{\bar{R}}_{i}^{t},{\bar{C}}_{i}^{t})=\text{Beta}(\mu |{\bar{R}}_{i}^{t}+a,{\bar{C}}_{i}^{t}-{\bar{R}}_{i}^{t}+b).$$

From Equations (4) and (6), we obtain

(7) $$P_{i}^{t+1}=\frac{{\bar{R}}_{i}^{t}+a}{{\bar{C}}_{i}^{t}+a+b}.$$

This model is a natural extension of FBM (Yu and Cohen, 2009) to the two-alternative choice task (for this reason, we will refer to our model as FBM). An alternative is repeatedly chosen while its predictive distribution is higher than those of the other due to the WTA strategy. Because the empirical probability of reward for an alternative converges to its baiting probability in repeated choices, *P*^*t*^_*i*_ gradually approaches to λ_*i*_ and the variance of *P*^*t*^_*i*_ decreases. As a result, FBM tends to choose exclusively the high payoff alternative after a large number of observations. Hence, the matching law [Equation (1)] is satisfied in *t* → ∞ because such a exclusive choice unboundedly increases both *R*^*t*^_*i*_ and *C*^*t*^_*i*_ of the high payoff alternative.

**Figure 1:**
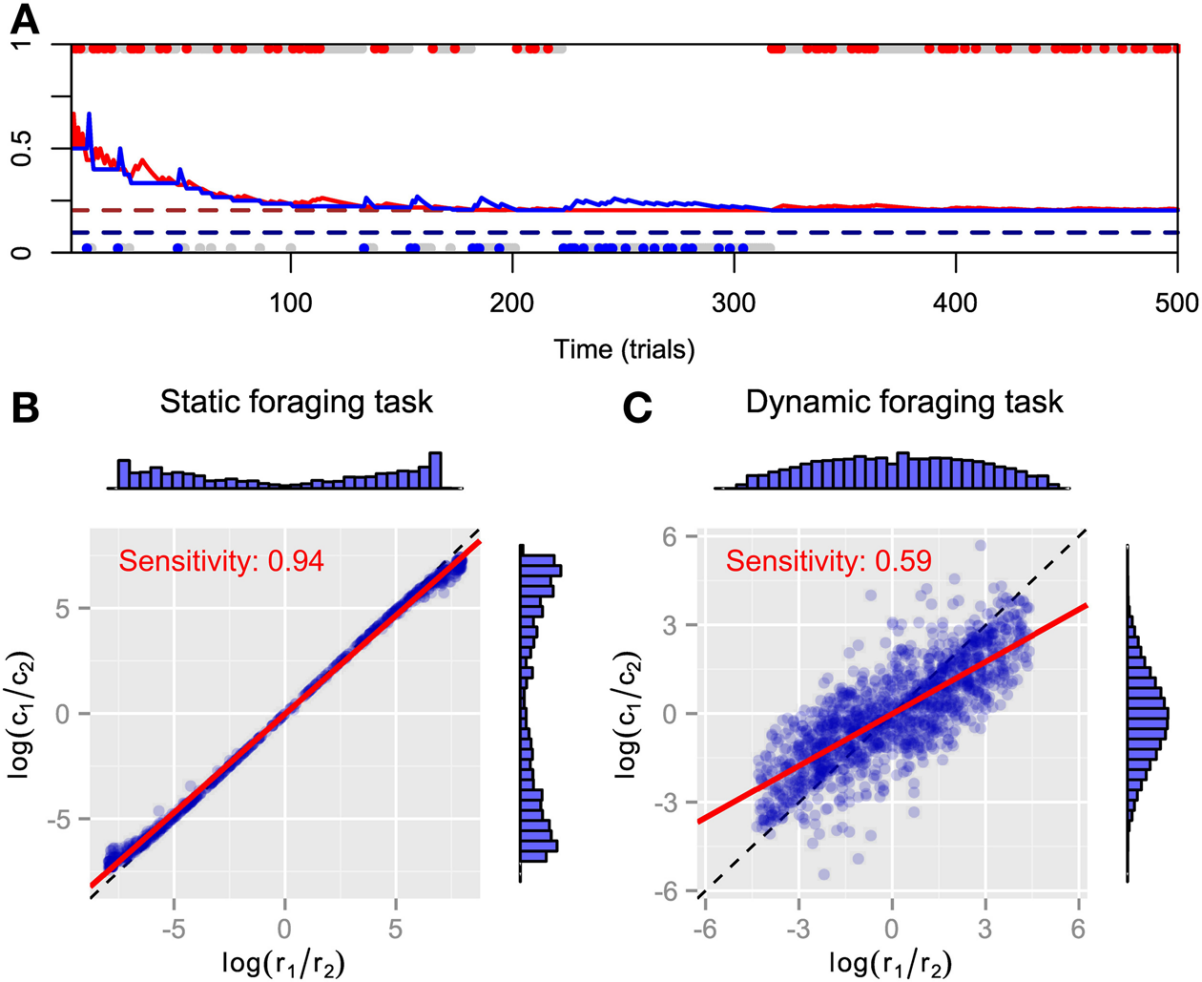
**Simulation results for FBM. (A)** Time course of predictive distributions for alternatives #1 (red solid line) and #2 (blue solid line) in static foraging task. Dashed lines indicate baiting probabilities of alternatives #1 (dark red) and #2 (dark blue). Upper and lower dots respectively represent choices for alternatives #1 and #2 in that trial and colored dots (red or blue) represent that the model received a reward at that trials. **(B)** Reward log ratios as a function of count log ratios in static and **(C)** dynamic foraging tasks. Blue symbols represent pairs of log ratios calculated in block where baiting probabilities are fixed and distributions of dots are represented by marginal histograms. Red line indicates best-fitted line to points and inner text shows its slope, i.e., sensitivity parameter of generalized matching law. Dashed line is identity line.

We simulated FBM in static and dynamic foraging tasks. The time course for the predictive distributions is shown in Figure 1A. As can be expected, both predictive distributions approach the respective baiting probabilities and FBM behavior converges to exclusive choice of the high payoff alternative in static foraging tasks. However, the steady-state choice behavior of animals in static concurrent VI schedules has not been thought to be exclusive (Baum, 1982; Davison and McCarthy, 1988; Baum et al., 1999). It might be that there are not enough trials for choice behavior to actually reach a steady state. Figures 1B,C plot the log ratios of rewards and choices in both tasks. The marginal histograms indicate the FBM's strong preference for the alternative that has the highest baiting probability, because most pairs of log ratios lie near the endpoints of the matching line. We found that bias is nearly zero and sensitivity is nearly one in the static foraging tasks (Figure 1B) by least-square fitting the generalized matching law [Equation (2)] to the data. Therefore, the model exhibits matching behavior in the static foraging tasks. However, the model no longer exhibits matching behavior in dynamic foraging tasks, a result that is inconsistent with the behavior of monkeys (Corrado et al., 2005) (Figure 1C). This can be because the model adheres to past experience and cannot adapt rapidly to changes in the environment.

### 2.2. Extended bernoulli estimators

One possible way of improving the model to enable it to rapidly adapt to changes in the environment is to introduce a forgetting mechanism for past rewards and choice history. We therefore assume a simple extended model, which utilizes only the *L* most recent rewards and choices for the estimates. Hence, the predictive distribution becomes

(8) $$P_{i}^{t+1}=\frac{(\sum_{l=0}^{L-1}r_{i}^{t-l})+a}{(\sum_{l=0}^{L-1}c_{i}^{t-l})+a+b}$$

We refer to this model as windowed FBM (WFBM).

Another possibility may be derived from the idea that humans and animals may innately believe their environment is volatile. Here, we propose a model that estimates time-varying reward probabilities. Although there are several ways to model a belief of a volatile environment, we assume our model believes that μ^*t*^_*i*_ remains unchanged with probability α, or else (with probability 1 − α) changes completely. This idea is derived from the dynamic belief model (DBM), proposed by Yu and Cohen as a model of sequential effect (Yu and Cohen, 2009). Our model is a natural extension of DBM to a two-alternative choice task. Thus, we refer to our model as DBM. The transition of μ^*t*^_*i*_ is modeled as a mixture of the posterior and prior distributions

(9) $$\begin{array}{l} p(\mu_{i}^{t+1}=\mu |R^{t},C^{t})=\alpha p(\mu_{i}^{t}=\mu |R^{t},C^{t})\\ \;+(1-\alpha )\text{Beta}(\mu |a,b), \end{array}$$

where 0 ≤ α ≤ 1 represents the model's expectations of the stability of the environment. However, the posterior distribution is no longer a Beta distribution:

(10) $$\begin{array}{l} p(\mu_{i}^{t}=\mu |R^{t},C^{t})\\ =p{\left(\mu_{i}^{t}=\mu |r_{i}^{t},c_{i}^{t}=1,R^{t-1},C^{t-1}\right)}^{c_{i}^{t}}p{\left(\mu_{i}^{t}=\mu |R^{t-1},C^{t-1}\right)}^{1-c_{i}^{t}}\\ ={\left[{\left(\frac{p(r_{i}^{t}=1|\mu_{i}^{t}=\mu )}{p(r_{i}^{t}=1|R^{t-1},C^{t-1})}\right)}^{r_{i}^{t}}{\left(\frac{p(r_{i}^{t}=0|\mu_{i}^{t}=\mu )}{p(r_{i}^{t}=0|R^{t-1},C^{t-1})}\right)}^{1-r_{i}^{t}}\right]}^{c_{i}^{t}}\\ p(\mu_{i}^{t}=\mu |R^{t-1},C^{t-1})\\ ={\left[{\left(\frac{\mu }{P_{i}^{t}}\right)}^{r_{i}^{t}}{\left(\frac{1-\mu }{1-P_{i}^{t}}\right)}^{1-r_{i}^{t}}\right]}^{c_{i}^{t}}p(\mu_{i}^{t}=\mu |R^{t-1},C^{t-1}), \end{array}$$

where we use Equation (3). Then, predictive distribution *P*^*t*^_*i*_ is calculated with Equations (4), (10), and (11). Note that these models are equivalent to FBM when *L* → ∞ and α = 1.

**Figure 2:**
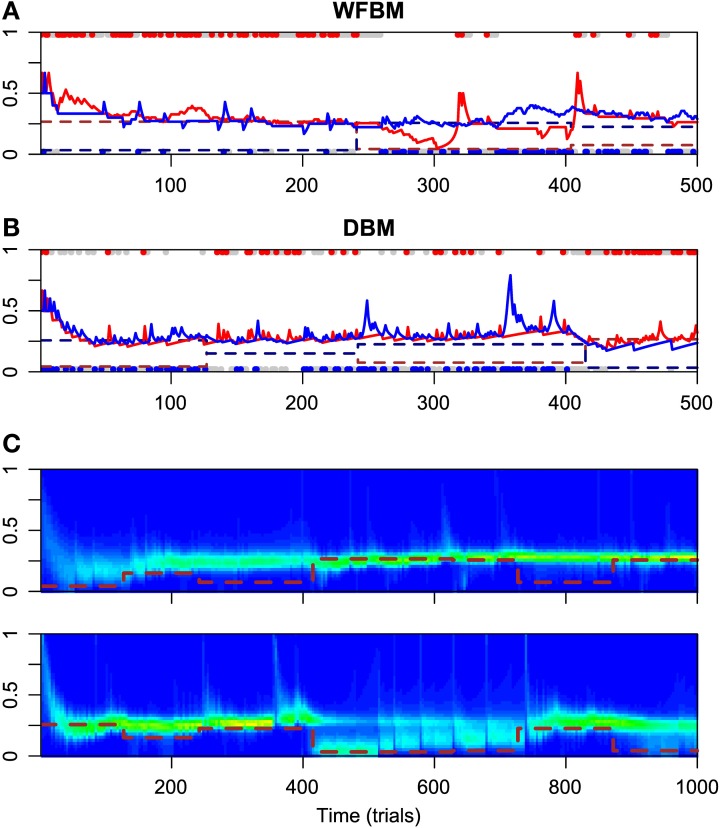
**Simulation results for WFBM and DBM in dynamic foraging task**. Simulation parameters were set to *L* = 60 and α = 0.99. **(A)** Time course of predictive distributions of WFBM and **(B)** DBM. Details in figure are described in caption of Figure 1A. **(C)** Time course of posterior distributions of DBM for reward probabilities of alternative #1 (top) and #2 (bottom). Brown dashed lines are baiting probabilities for respective alternatives.

**Figure 3:**
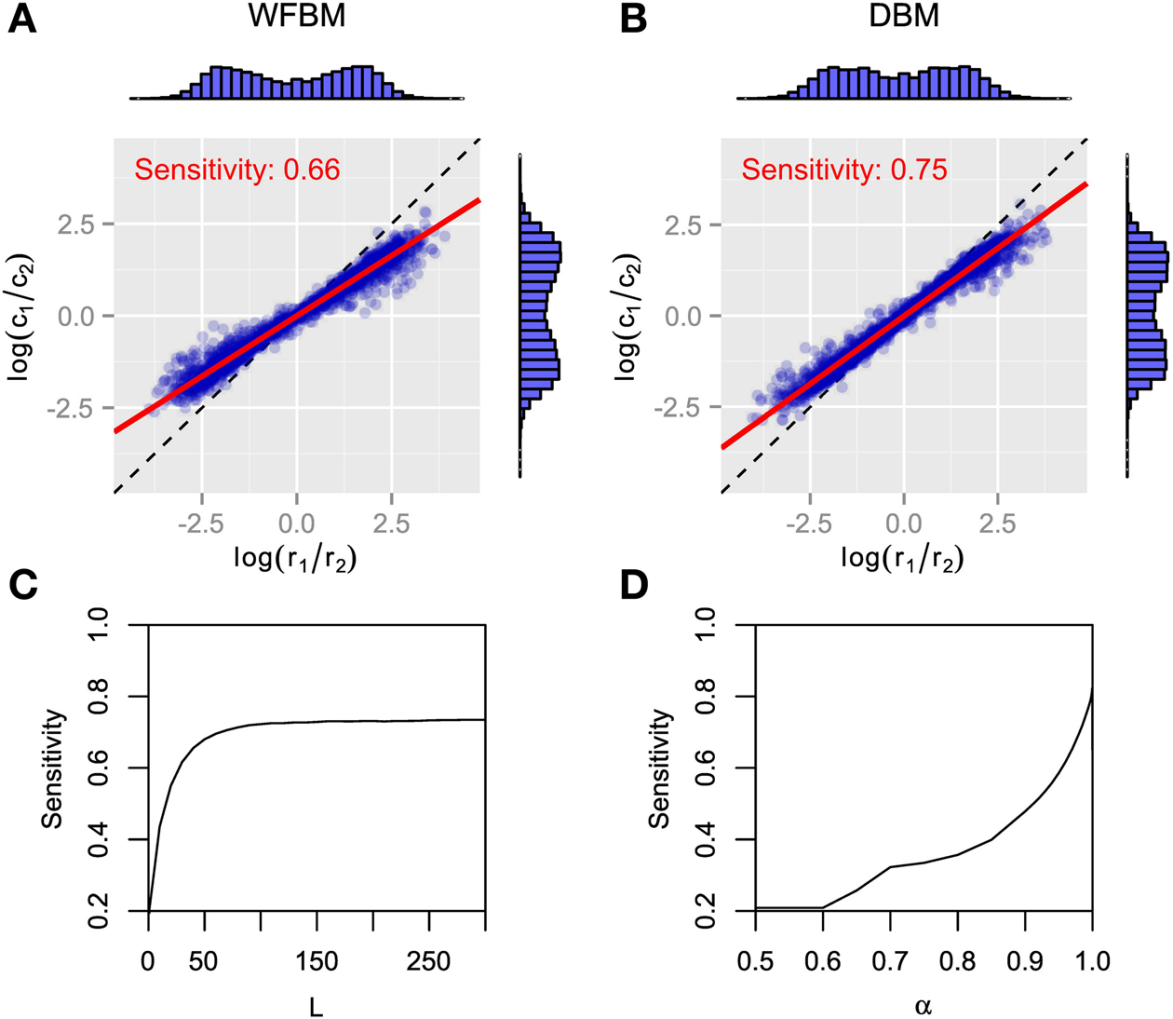
**Analytical results for matching behavior of WFBM and DBM in dynamic foraging tasks**. **(A,B)** Reward log ratios as a function of count log ratios. Details in figure are described in caption of Figures 1B,C. Simulation parameters were set to *L* = 40 and α = 0.99. **(C,D)**Sensitivity as a function of parameters of WFBM and DBM.

Figure 2 has the time courses for the predictive distributions of WFBM and DBM, and the posterior distributions of DBM in the dynamic foraging task. Neither model is stuck on one alternative and can follow the changes in schedules as expected. There is a clear difference in the predictive distribution trajectories. Because WFBM exploits recent samples, its predictive distribution for the unchosen alternative can approach the true baiting probability. DBM's predictive distribution for the unchosen alternative, on the other hand, is only retracted to the mean of the prior, i.e., 0.5. Both models demonstrate matching behaviors even in the dynamic foraging task (Figure 3). More precisely, the behaviors slightly deviate from the matching law toward an unbiased choice. This phenomenon is known as *undermatching* (Baum, 1979). Because the models' parameters *L* and α control the effect of past experience, the degree of undermatching is controlled by the parameters. The sensitivities that were fitted in the experiments were in a range of about 0.44 to 0.91 (Hinson and Staddon, 1983; Corrado et al., 2005; Lau and Glimcher, 2005). Hence, we basically focused on parameter regions 10 ≤ *L* and 0.9 ≤ α.

**Figure 4:**
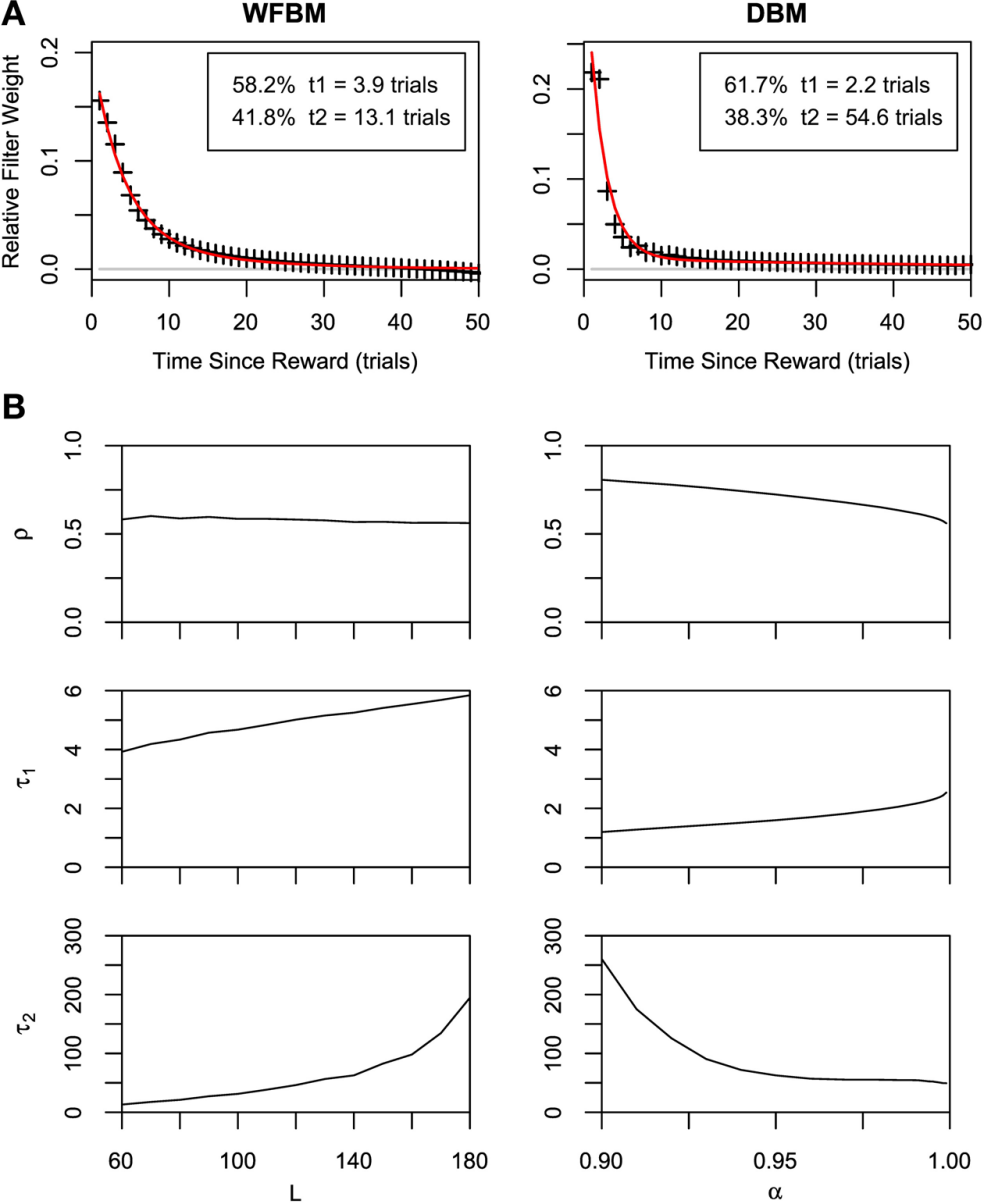
**Results of Wiener-Hopf analysis for WFBM and DBM in dynamic foraging task**. **(A)** Symbols represent normalized Wiener-Hopf kernel and red line represents best fitted double-exponential filter. Double-exponential filters are better fitted to data than single-exponential filter (likelihood ratio test, *p* « 0.001). Insets show time constants for each exponential component and their combining rate. Simulation parameters were set to *L* = 60 and α = 0.99. **(B)** Fitted parameters of double-exponential filter ρ, τ_1_, and τ_2_ to simulation data of WFBM (left column) and DBM (right column). Abscissas represent parameters of WFBM or DBM.

The dependence of choices on reward history has been studied in several monkey experiments. An exponential shaped dependency was first reported (Sugrue et al., 2004) and then heavier-tailed dependencies were reported (Corrado et al., 2005; Lau and Glimcher, 2005). We tested our models by calculating the dependence of choices on reward history (Figure 4A). Suppose that dependency is expressed with a linear filter kernel κ(*i*) as in previous studies. The kernel is calculated by minimizing the following Wiener-Hopf equation,

(11) $$\frac{1}{2}\sum_{t}{\left[\left(c_{1}^{t}-c_{2}^{t})-\sum_{i=1}^{K}\kappa (i)(r_{1}^{t-i}-r_{2}^{t-i}\right)\right]}^{2}.$$

Then, we fit the exponential filter and double-exponential filter that were introduced by Corrado et al. (2005) to the normalized kernel:

(12) $$\begin{array}{l} \epsilon_{1}(i)=\frac{\exp(-i/\tau_{0})}{\sum_{k=1}^{K}\exp(-k/\tau_{0})},\\ \epsilon_{2}(i)=\rho \;\frac{\exp(-i/\tau_{1})}{\sum_{k=1}^{K}\exp(-k/\tau_{1})}+(1-\rho )\frac{\exp(-i/\tau_{2})}{\sum_{k=1}^{K}\exp(-k/\tau_{2})},\;\;\;\;\;\; \end{array}$$

where τ_0_ and τ_1_ ≤ τ_2_ are time constants and 0 < ρ < 1 is the combining rate. Note that ϵ_2_ is identical to ϵ_1_ when τ_1_ = τ_2_. The double-exponential filter is rather more well-fitted than the single one for WFBM and DBM (likelihood ratio test, *p* « 0.001; adjusted *r*^2^ for double and single exponential filters are 0.99 and 0.98 for WFBM, and 0.94 and 0.85 for DBM). The kernel for WFBM has a negative value around *L* but it disappears if *L* is much longer than *K*. The kernel for DBM drops sharply and decays slowly. The sharp drop probably arose from the exponential decay of reward history, which is embedded in the posterior distributions [Equation (10)]. Because a decision is made due to the difference in two predictive distributions and both distributions decay at the same rate, the effect of one predictive distribution would have persisted slightly longer and hence the kernel included a longer exponential component. This characteristic is qualitatively consistent with the experimental results Corrado et al. (2005). The fitting parameters for the two monkeys in Corrado et al. (2005) were ρ = 0.4, τ_1_ = 2.2, and τ_2_ = 17.0 (monkey F), and ρ = 0.25, τ_1_ = 0.9, and τ_2_ = 12.6 (monkey G). Although there were no suitable WFBM and DBM parameters that exactly matched their fitting parameters to those of the monkeys, similar values were obtained for smaller *L* and larger α (Figure 4B).

**Figure 5:**
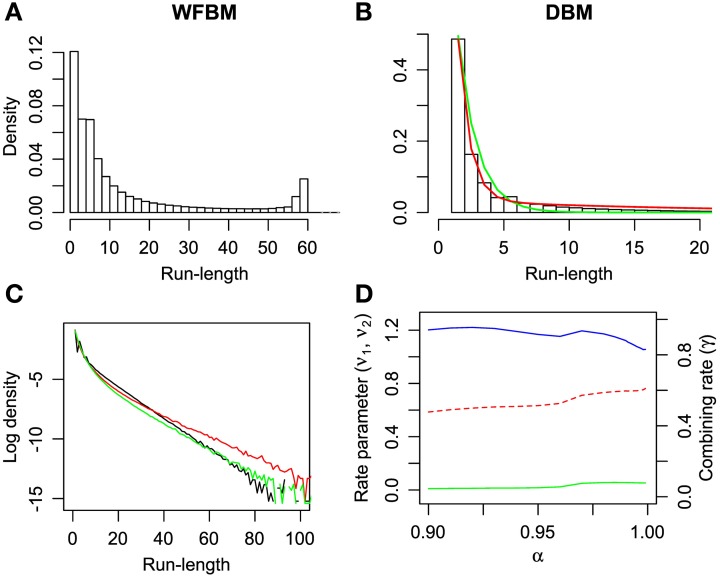
**Run-length distributions of windowed FBM and DBM in dynamic foraging task**. Simulation parameters were set to *L* = 60 and α = 0.99. **(A,B)** Bars represent densities of run length for alternative #1. Single (green line) and double-exponential (red line) functions fitted to run-length distributions of DBM. Double-exponential function is fitted better than single one (likelihood ratio test, *p* « 0.001). **(C)** Log probability density of run-length distribution of DBM (black line) and linear-nonlinear Poisson models (red and green lines) that are fitted to monkeys' experimental data in Corrado et al. (2005). **(D)** Fitted parameters of double-exponential function with different values of α. Left ordinate indicates value of rate parameters ν_1_ (green line) and ν_2_ (blue line), and right indicates value of combining rate γ (red line).

It is known that the probability of switching alternatives is nearly constant against the number of consecutive choices for one alternative (run length) in the concurrent VI schedule (Heyman and Luce, 1979). Hence, run lengths are distributed exponentially but, in a dynamic foraging task, the distribution seems to be a mixture of exponentials (Corrado et al., 2005). The distribution of WFBM does not monotonically decrease and there is a peak where the run length is nearly equal to *L*. Therefore, the distribution is neither an exponential nor a mixture of exponentials. This nature is consistent on different values of *L*. However, DBM demonstrates an exponential like distribution. We fitted single and double exponential functions,

(13) $$\begin{array}{l} \phi_{1}(l)=\nu_{0}\exp(-\nu_{0}(l-1)),\\ \phi_{2}(l)=\gamma \nu_{1}\exp(-\nu_{1}(l-1))\\ \;+(1-\gamma )\nu_{2}\exp(-\nu_{2}(l-1)), \end{array}$$

to the distribution, where *l* ≥ 1 is the run length, ν_0_ and ν_1_ < ν_2_ are the rate parameters and γ is the combining rate. The distribution is well-fitted by the double exponential function (Figure 5B; likelihood ratio test, *p* « 0.001; *r*^2^ for the double and single exponential functions are 0.99 for the former and 0.96 for the latter). The run-length distribution in monkey experiments has few frequencies of a very short run length; however our models have the largest frequency at the run length of 1 (Figures 5A,B). This difference can be due to the absence of change-over-delay (COD) in our schedule. If our model had and exploited prior knowledge about COD as well as the proposed model for the previous experiment (Corrado et al., 2005), the frequency at a run length of 1 could disappear. We simulated linear-nonlinear-Poisson (LNP) models that were fitted to the monkeys' experimental data in Corrado et al. (2005) and compared run-length distributions (Figure 5C). Note that COD was not considered for the LNP models that was different from Corrado et al.'s approach Corrado et al. (2005). Because the absence of COD could affect the occurrence of short run lengths, log probability densities were compared to count differences at long run lengths. The calculated mean squared differences of DBM against LNP models for two monkeys corresponded to ~0.67 and 0.16. The double-exponential function is better than the single one in different α and the fitted parameters are slightly affected by α (Figure 5D).

#### 2.2.1. Harvesting performance

**Figure 6:**
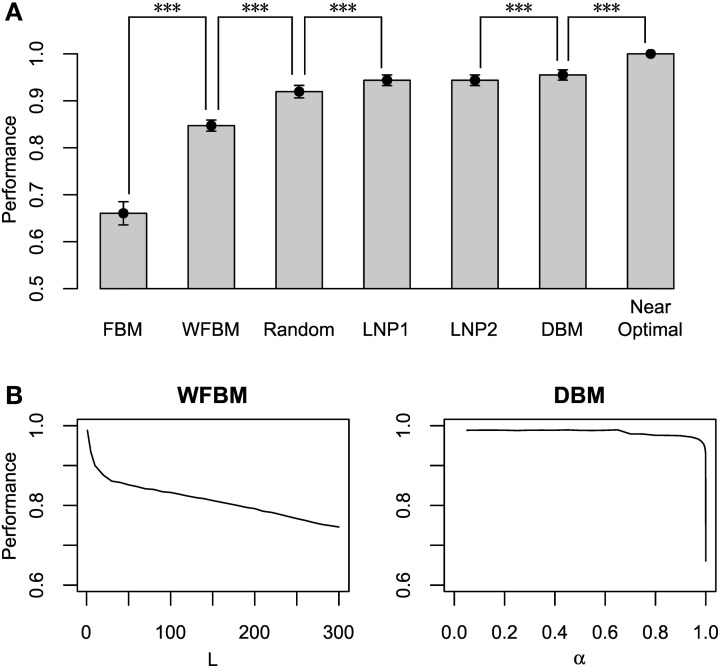
**Normalized harvesting performance of each model**. **(A)** Average normalized total rewards earned by each model divided by average total rewards of near-optimal model. Near-optimal model uses strategy that maximizes average total rewards proposed by Sakai and Fukai (2008a) with previous knowledge on details of schedule. Error bars indicate standard deviations around mean. Simulation parameters were set to *L* = 60 and α = 0.99. **(B)** Harvesting performance of WFBM and DBM as a function of their parameters. ^***^p « 0.001.

Figure 6A compares the harvesting performance of the models, which is normalized by the performance of a near-optimal probabilistic decision making model. The near-optimal model knows the details of the schedules, i.e., both the baiting probabilities and the change points. It distributes its choices according to the choice probabilities that on average maximize the total reward (Sakai and Fukai, 2008a). Due to such given knowledge, none of the other models can exceed the performance of the near-optimal model. We carried out paired *t*-tests between the models, in which the means of total reward for an identical schedule were paired. The FBM and WFBM (*L* = 60) are more inferior than the random choice model that chooses by tossing an unbiased coin. The DBM (α = 0.99) outperforms FBM, WFBM, and LNP models (*p* « 0.001) but the differences from the LNP models are very small. Harvesting performance is less when a model memorizes a more distant past (Figure 6B).

## 3. Discussion

We demonstrated that deterministic Bayesian decision making models can account for the matching law. We confirmed that a simple Bernoulli estimator with a deterministic decision policy demonstrated matching behavior in a static foraging task. We also studied an extended model that includes a belief about a changing environment. The belief effectively works to wipe out the past experience of the model and hence the model can capture three characteristics of behaviors observed in the experiments. First, our model accounts for undermatching, which is a well-known phenomenon in which choices deviate slightly from the matching law (Baum, 1974, 1979; Sugrue et al., 2004). Several studies have addressed possible causes of undermatching, i.e., limitations in the learning rule (Soltani and Wang, 2006), mistuning of parameters (Loewenstein, 2008), and diffusion of synaptic weights (Katahira et al., 2012). This study suggested the cause from a computational perspective, i.e., undermatching was the consequence of a belief in environmental volatility. Second, our model exhibits double-exponential shaped reward history dependency. This is consistent with recent monkey experiments (Corrado et al., 2005; Lau and Glimcher, 2005). Third, the run-length distribution of our model is better fitted by a double-exponential function than a single exponential function. This is also consistent with the previous study (Corrado et al., 2005) although our task did not include changeover delay, which can strongly affect the frequency of shorter run lengths. Quantitatively validating our model such as checking its goodness of fit to raw experimental data would be worthwhile.

The previous models implicitly or explicitly use the strategy of probabilistic choice selection and they learn the choice probability of respective alternatives that satisfy the matching law (Corrado et al., 2005; Lau and Glimcher, 2005; Loewenstein and Seung, 2006; Soltani and Wang, 2006; Sakai and Fukai, 2008a; Simen and Cohen, 2009). Such probabilistic models use a scaling parameter that maps internal decision variables to appropriate choice probabilities and the parameter generally requires fine-tuning (Soltani and Wang, 2006; Fusi et al., 2007). In contrast, as our models act deterministically according to decision variables, no tuning is required for a parameter at the decision stage.

We argued that matching behavior can be explained by a deterministic choice strategy at the computational level. Loewenstein and Seung (2006) proposed biologically inspired synaptic learning rules for neural networks at the neural implementation level. They proved that neural networks developed by covariance-based learning with the assumption of a low learning rate demonstrated matching behaviors. However, this assumption causes the choice to be affected by relatively distant past rewards and the kernel for reward history dependency consequently flattens. A more microscopic spiking neural network model, in which double-exponential dependency in foraging tasks is demonstrated, has been proposed (Soltani and Wang, 2006). However, there is a huge gap between the computational principles of our deterministic macroscopic models and their stochastic microscopic model. This gap can be filled by using a method of reducing spiking neuron models to the diffusion equation (Roxin and Ledberg, 2008). There have been some other neural network models that can show heavy-tailed dependency of choices on past experience. A reservoir network (Jaeger et al., 2007), which can reproduce neural activity in the monkey prefrontal cortex, preserves the memory trace of a reward with one or two time constants (Bernacchia et al., 2011). The composite learning system of faster and slower components is flexible to abrupt changes in the environment (Fusi et al., 2007). These models could be a possible neural implementation for our model. Furthermore, our models are an extension of that by Yu & Cohen who argued that decision variables of their model can be approximated by a linear exponential filter, and that there are neural implementations for that operation (Yu and Cohen, 2009).

Because matching behavior often deviates from optimal behavior in the sense of total reward maximization (Vaughan, 1981), it is not likely to be a consequence of optimization. However, our model acts optimally in terms of Bayesian decision making with an incorrect assumption about the environment, indicating that matching behavior is a bounded optimal behavior. This idea is consistent with the theory of Sakai and Fukai (2008b) who found any learning method neglecting the effect of a choice on future rewards displays matching behavior if choice probabilities are differentiable with respect to parameters (Sakai and Fukai, 2008b). Note that the choice probabilities of our model are not differentiable. Hence, we confirmed that their theory could be correct in such extreme cases.

## 4. Materials and methods

### 4.1. Details of simulation

The reward schedule is analogous to the experiment by Corrado et al. (2005). We randomly set the baiting probabilities that satisfied λ_1_ + λ_2_ = 0.3 and their ratios were 1:8, 1:6, 1:3, 1:2, 1:1, 2:1, 3:1, 6:1, and 8:1 in a static setting. There were 10,000 trials in the simulations. The baiting schedule in the dynamic setting was divided into blocks, in which the baiting probabilities were fixed, and their sum and ratios were the same as those in the static setting. The block length was uniformly sampled from [50, 300] and there were 300 blocks in the simulations. We did not include change-over-delay (COD), i.e., the cost to switch from one alternative to another, which was different from Corrado et al. (2005). The hyper-parameters were set to *a* = 1 and *b* = 1 in all the simulations.

### Conflict of interest statement

The authors declare that the research was conducted in the absence of any commercial or financial relationships that could be construed as a potential conflict of interest.
